# Antibacterial effect of resveratrol extract compared to chlorhexidine mouthwash against primary cariogenic pathogen, Streptococcus mutans

**DOI:** 10.4317/jced.61456

**Published:** 2024-07-01

**Authors:** Maryam Pournasir, Fereshteh-Naser Alavi, Reza-Tayefeh Davalloo, Hadi-Sedigh Ebrahim-Saraie, Mohammad-Ebrahim Ghaffari

**Affiliations:** 1 Dentist, Tehran, Iran; 2 Associate professor, Dental Sciences Research Center, Department of Operative Dentistry, School of Dentistry, Guilan University of Medical Sciences, Rasht, Iran; 3 Assistant professor, Razi Clinical Research Development Unit, Razi Hospital, Guilan University of Medical Sciences, Rasht, Iran; 4 Assistant professor, Department of Biostatics and Epidemiology, School of Health, Qom University of Medical Sciences, Qom, Iran

## Abstract

**Background:**

Modern dental caries prevention methods have focused on using herbal products that ideally inhibit the critical cariogenic bacteria (*Streptococcus mutans*). The present study compared antibacterial efficacy of the resveratrol herbal extract and 0.12% chlorhexidine (CHX) against Streptococcus mutans (*S. mutans*).

**Material and Methods:**

In this *in vitro* study the pure powder of resveratrol (Bulksupplement) was dissolved in dimethyl sulfoxide as its solvent to produce a 1000-µg/mL concentration of resveratrol solution, which was later used at different dilutions. The antibacterial effects of resveratrol solution and 0.12% CHX mouthrinse on the standard strain of *S. mutans* were determined using a minimum inhibitory concentration (MIC) test in a tube, minimum bactericidal concentration (MBC) test in a solid medium, and a well diffusion test to measure the zone of inhibition. The data were analyzed using Kruskal-Wallis, Bonferroni, and Man-Whitney tests (α=0.05).

**Results:**

The MIC and MBC of resveratrol was 250 µg/mL. In addition, this extract exhibited a diameter of 6.67 mm for the inhibition zone at only the 1000-µg/mL concentration. The MIC of CHX was 15.6 µg/mL, and its MBC was 31.25 µg/mL. The highest growth inhibition zone of CHX was 16.67 mm.

**Conclusions:**

Resveratrol extract exhibited a dose-dependent antibacterial (bacteriostatic and bactericidal) activity against *S. mutans*. Although it was not as effective as CHX, it might be a suitable alternative to prevent dental caries.

** Key words:**Antibacterial agents, Chlorhexidine digluconate, mouthwashe, resveratrol, streptococcus mutans.

## Introduction

Dental caries is still a significant orodental health problem in many countries. While dental caries is a problem in underprivileged and underdeveloped regions, it is also prevalent in developed societies (1).

The oral cavity is a complex ecosystem with many microbial factors, including viruses, bacteria, and fungi found in thin layers on oral surfaces such as teeth, the tongue, and mucosal surfaces, called biofilms. Frequent acidification of dental biofilms contributes to the emergence of acidogenic (capacity to produce organic acids) and aciduric (ability to survive under low pH conditions) microflora, including *Streptococcus mutans* (*S. mutans*) and *Lactobacillus* species. These bacteria rapidly ferment dietary carbohydrates, decreasing pH, which results in the demineralization and progressive destruction of the tooth structure. *S. mutans* is a gram-positive facultative anaerobic bacterial known as the primary etiologic agent of dental caries (1).

Currently, there are significant concerns about prevention and dental care in relation to dental caries. Mechanical oral hygiene methods include brushing and flossing are the dentists’ focus of attention to decrease microorganism counts, especially *S. mutans*. However, mechanical methods cannot eliminate oral biofilms in many patients. Malpositioned teeth or orthodontic appliances might make it difficult to control microbial plaque mechanically. Therefore, antimicrobial agents (chemical and herbal) have been recommended to improve the efficacy of mechanical methods. These agents, including mouthwashes, have a key role as supplements to daily homecare methods to prevent and control dental plaque (2-4).

Previous studies have shown the positive effects of mouthwashes containing active ingredients such as chlorhexidine (CHX) and essential oil on inhibiting *S. mutans* and controlling microbial plaque (5). Chlorhexidine digluconate is the gold standard in inhibiting plaque formation and has been considered a positive control in many studies to compare the effects of other agents (6). However, despite its advantages, it has disadvantages, including changing taste sensation, discoloration of teeth and xerostomia (7). These side effects have prompted research efforts to find more suiTable alternatives.

Recently, ever-increasing attention has been directed toward natural and herbal products as antibacterial agents to decrease the dental caries rate; the effects of most of these agents are dose-dependent (8,9). These agents have widespread biochemical properties and are classified into different categories: trepenoids, Tannins, stilbenes, flavonoids, anthraquinones, phenolic acids, and alkaloids (10). Resveratrol (RSV) (3,5,4’-trihydroxy-trans-stilbene) is a compound found in many herbal extracts, such as grapes, peanuts, and cranberry. It has several functions, including antibacterial, antiviral, antioxidative, anti-inflammatory, and anticancer effects (11). It may modify the bacterial virulence qualities resulting in decreased toxic substance production, biofilm inhibition, motility reduction, and quorum sensing disturbance (12).

RVS is usually extracted using maceration, ultrasound, and spectrometric methods, and pharmaceutical companies have also prepared its pure extract (13,14).

Osamudiamen *et al*. recently showed, as a first *in vitro* study on the antimicrobial activity of RSV and piceatannol polyphenols, these materials’ potent anticaries, antioxidative, and cytotoxic activity (14). At the same year, Li *et al*. showed resveratrol’s ability to decrease acid production, increase acid resistance, produce polysaccharides, and prevent biofilm formation of *S. mutans* at concentrations below the minimum inhibitory concentration (MIC) (15).

Based on the explanations above, inhibiting *S. mutans* can effectively prevent dental caries. Considering the various side effects of chemical mouthwashes, it is necessary to develope alternative mouthwashes containing natural products with antibacterial properties and minimal side effects. Since sufficient data are unavailable, the present *in vitro* study was undertaken to evaluate the effect of RSV as a mouthwash on inhibiting *S. mutans* growth and compare it with 0.12% CHX mouthwash.

## Material and Methods

The protocol of the present study was approved by the Ethics Committee of Guilan University of Medical Sciences (IR.GUMS.REC.1400.453). This *in vitro* study compared the antibacterial effects of resveratrol extract (Bulksupplements, USA) and 0.12% CHX mouthwash (Iran, Behsa) on *S. mutans*. Its pure extract powder was dissolved in dimethyl sulfoxide as a solvent to prepare the RSV solution, and its 1000-µg/mL concentration was achieved.

-The test microorganism

The studied bacteria was the standard strain of *S. mutans* (ATCC 35668), which was procured in its lyophilized form from the Iranian Center of Biological and Genetic Research. To prepare a proper concentration of the bacterial species for the test, the standard strain was inoculated into 5 mL of tryptone soy broth and incubated at 37ºC for 24 hours. A bacterial suspension equal to 0.5 McFarland standard (1.5×108 CFU/mL) was prepared from the one-day culture of the strain in Mueller-Hinton culture medium and used for subsequent tests.

-Determining the minimum inhibitory concentration (MIC)

The microbroth dilution method was used to determine MIC and the sensitivity of *S. mutans* to RSV and CHX solution at serial concentrations of the initial solution. First, 100 µL of Mueller-Hinton broth was prepared in the wells of a 96-well cell culture plate. Then, 10 µL of the diluted microbial suspension, equivalent to 0.5 McFarland concentration, was added to each well. Subsequently, 100 µL of 1000-µg/mL concentration of resveratrol solution was added to the first well (the final concentration of resveratrol in the first well reached 500 µg/mL); then, serial dilutions were prepared by removing 100 µL of the solution from the first well and transferring it to the next well. 100 µL of the solution was discarded from the final well. The same procedures were carried out for the CHX mouthwash. It should be pointed out that one column was considered the positive control, i.e., it contained the culture medium and bacteria. One column was considered negative control; i.e., it contained only the culture medium (to ensure no contamination of the culture medium), and one column contained only the test material (to ensure no contamination). Then the plate was transferred into an incubator at 37ºC and the results were evaluated after 24 hours to determine MIC by observing turbidity of tube. Turbidity indicated bacterial growth, and no turbidity indicated no bacterial growth. The minimum concentration of the test solution that inhibited bacterial growth was recorded as the solution’s MIC.

-Determining minimum bacterial concentration (MBC)

To determine MBC, some solution from each tube, irrespective of turbidity or clearness, was cultured on a plate of solid blood-agar culture medium. After 24 hours of incubation at 37ºC, the minimum concentration of the solution at which no bacterial growth was observed was considered MBC. MIC and MBC tests were repeated three times, and the most frequent responses were recorded.

-Determining antibiotic sensitivity using the well diffusion method

First, the available standard strain colonies of *S. mutans* under study were cultured in the Mueller-Hinton blood agar culture medium (Merck, Germany). Then several colonies were taken from the one-day culture and transferred to tubes containing sterile physiologic serum. The tubes were incubated at 37ºC for 30 minutes to prepare a suspension with 0.5 McFarland turbidity. A sterile swab was dipped in the tube and pressed hard against the inner walls of the tube to remove the excess suspension, followed by culturing on the Muller-Hinton agar containing 5% blood in all directions (spreadsheet culturing) for complete inoculation on the plate surface. The plate was kept at room temperature for 5-10 minutes to remove its moisture. A special sterile puncher was used to produce wells at 20-mm distances from the plate edge, 25 mm apart from each other, on the plate surface. Then, 20 µL from each solution (RSV / CHX) was added to each well at concentrations of 125, 250, 500, and 1000 µg/mL) using a sterile sampler. Finally, the plates were incubated at 37ºC for 24 hours. Then the efficacy of the two solutions was compared based on the diameter of the growth inhibition zone using a mm-graduated ruler. The solvent was used as the negative control. The procedures were repeated three times for accuracy.

-Statistical analysis

The well diffusion test data were analyzed with SPSS 26. The data were analyzed with the Shapiro-Wilk test, and since the data were not distributed normally, the Kruskal-Wallis test, multiple comparisons with Bonferroni corrections, and Mann-Whitney tests were used to compare the growth inhibition zone diameters between the two solutions and different concentrations. Statistical significance was set at *P*<0.05.

## Results

-MIC and MBC assessments

The MIC and MBC of RSV extract for *S. mutans* was 250 µg/mL. However, the MIC and MBC of 0.12% CHX were 15.6 and 51.25 µg/mL, respectively, which were significantly less than that of RSV ( Table 1).

-Well diffusion assessment

 Table 2 presents the growth inhibition zone diameters for *S. mutans* at different concentrations of CHX and RSV. Based on the results, RSV exhibited a bacterial growth inhibition zone only at the pure concentration of 1000 µg/mL.

Kruskal-Wallis test showed the significant effects of different concentrations of 0.12% CHX on the growth inhibition zones of *S. mutans* (*P*=0.017). Two-by-two comparisons showed that only the 1000-µg/mL concentration of CHX was significantly different from the 125-µg/mL concentration (*P*=0.009). However, other comparisons did not reveal significant differences. In addition, different concentrations of RSV extract significantly affected the growth inhibition zone diameters (*P*=0.013). Two-by-two comparisons showed that the 1000-µg/mL concentration significantly differed from its lower concentrations (*P*=0.04) ( Table 2).

Comparisons of growth inhibition zones between RSV and CHX at 125, 250, 500, and 1000 µg/mL concentrations showed significant differences between the two materials (*P*=0.03, *P*=0.03, *P*=0.02 and *P*=0.04 respectively; Mann-Whitney test).

## Discussion

Dental caries is one of the most prevalent mouth infectious diseases and a major problem for the world population (15). The main strategy to prevent and treat dental caries is controlling the activity of and inhibiting *S. mutans* gram-positive cocci (16).

The present study evaluated and compared the inhibitory effects of RSV herbal extract and 0.12% CHX on the growth of the standard strain of *S. mutans*. The results showed that the RSV solution had a favorable inhibitory effect on *S. mutans* but was less effective than CHX. According to previous research, CHX is the gold standard in the local chemical treatment against oral microorganisms, including *S. mutans* (6), consistent with the present study, in which CHX exhibited high antibacterial potential with an MIC of 15.6 µg/mL and an MBC of 31.25 µg/mL for *S. mutans*. In addition, at the maximum concentration, it had a growth inhibition zone diameter of 16.67 mm, with higher growth inhibition zone diameters at lower concentrations, indicating this material’s high antibacterial potential. A study showed a very high antibacterial potential for CHX, with no antibacterial activity only at a very low concentration (0.0005%) (17). CHX is a biguanide adsorbed to the tooth and mucosal surfaces; its antibacterial activity is attributed to the absorption by extracellular polysaccharides. However, using CHX is associated with some side effects, including tooth and tongue discoloration, taste loss, and xerostomia (5).

Modern dental caries prevention methods have focused on natural herbal products such as polyphenols (18). Polyphenols are one of the main ingredients of plants, with antioxidative, anti-inflammatory, and antibacterial activity (19). Polyphenols are categorized into phenolic acid, flavonoid, and stilbene subgroups. Resveratrol is a kind of stilbene with a chemical formula of 3,5,4’-trihydroxy stilbene and is found in different plants. It has shown efficacy in health and has anticancer, anti-inflammatory, antidiabetic, and antimicrobial activities (20-23).

In the present study, the powder of the pure extract of RSV (Bulksupplements) was used to produce its solutions. The results showed favorable antibacterial effects (bacteriostatic and bactericidal) on the standard strain of *S. mutans*. Its MIC and MBC was 250 µg/mL, with the highest growth inhibition zone diameter of 6.67 mm at a concentration of 1000 µg/mL. However, the lower concentration of this material did not show growth inhibition zone for *S. mutans*, indicating the dose-dependent efficacy of RSV extract.

The present study results are consistent with previous studies. In one study, the compounds extracted from the root of Polygonum cuspidatum, including physcion, emodin, and resveratrol extracts, destroyed the infection factors of *S. mutans* (24). Osmudiamon *et al*. (14) reported the anticaries and antioxidative activity of polyphenols extracted from Mezoneuron benthamianum plant species, including resveratrol (stilbene), piceatannol (stilbene), and gallic acid (benzoic acid) against *S. mutans* and several other oral bacteria. RSV had the highest anticaries and antioxidative activity of the extracts above, with an MIC of 25 µg/mL. Although the MIC in that study was lower than that in the present study, its antibacterial activity against *S. mutans* was confirmed.

Nijampatnam *et al*. (16) showed that piceatannol extract with low micromolar concentration (52 µmol) selectively inhibited the formation of *S. mutans* biofilms, with no inhibitory effect on other streptococcal species. In that study, using RSV and piceatannol decreased the dental caries rate with no cytotoxic effects in rats. Although RSV has exhibited antibacterial activity against gram-positive and gram-negative bacteria, it is more active against gram-positive bacteria such as *S. mutans*, possibly due to the single-layer cell wall of gram-positive bacteria (25). Some resveratrol derivatives show bactericidal activity against Gram-positive bacteria in the same low micromolar range of traditional antibiotics, with an original mechanism of action that combines membrane permeability activity with ionophore-related activities (26).

Li *et al*. (15) evaluated the antibacterial activity of the powder of RSV extract (Sigma Aldrich) and reported an MIC of 800 µg/mL against *S. mutans*, which was higher than that in the present study. In addition, in the study above, RSV at concentrations below MIC inhibited the pathogenic factors of *S. mutans*, including acid production, acid tolerance, the synthesis of extracellular polysaccharides, and biofilm formation in a dose-dependent pattern.

Although RSV’s precise antibacterial activity mechanism has not been elucidated, it might interfere with cell membrane proteins and inhibit bacterial enzymatic systems through its antiproliferative properties (27).

It is difficult to directly compare different studies due to variations in microbial evaluations, microbial species, the medicinal plants’ region of origin, the extract used, how the extracts are prepared, and other confounding parameters. Based on the studies mentioned above and the present study, RSV has favorable inhibitory effects on *S. mutans* gram-positive bacterial species. Although this effect was less than that of CHX, considering the unfavorable side effects of CHX, RSV might be considered a material with antibacterial potential. On the other hand, the safety of herbal extracts has been confirmed by studies (7,12). Therefore, the present study might be a precursor for future clinical studies, and attention to its results might help produce materials to prevent caries and preserve oral health.

In the present study, only the standard strain of *S. mutans* was evaluated, and other oral microorganisms and other bacteria involved in dental caries were not evaluated. It is suggested that future studies be carried out similar to clinical conditions to better determine the best dose with the least side effects.

## Conclusions

Under the limitations of the present study, it can be concluded that RSV extract had favorable antibacterial activity (bacteriostatic and bactericidal) against the standard strain of *S. mutans*. Although its efficacy was less than 0.12% CHX mouthrinse, but it can be a suiTable alternative product to control dental caries.

## Figures and Tables

**Table 1:** Minimum Inhibitory Concentration and Minimum Bactericidal Concentration values (µg/ml).

Agent	MIC	MBC
Resveratrol	250	250
Chlorhexidine	15/6	31/25

**Table 2:** The mean ± SD of inhibition zone of streptococcus mutans in different concentration of agents.

Agent	Concentration(µg/ml)	Inhibition zone(mm)	P-value
Chlorhexidine	125 ^a^ 250 ^ab^ 500 ^ab^ 1000^b^	10/67±1/55 14/67±0/58 15±0 16/67±1/55	0/017*
Resveratrol	125^a^ 250^a^ 500^a^ 1000^b^	0±0 0±0 0±0 6/67±1/15	0/013*

*Kruskal-wallis test For each agent, dissimilar letters means significant difference between various concentrations (*p*<0.05).

## Data Availability

The datasets used and/or analyzed during the current study are available from the corresponding author.

## References

[1] Downer MC, Drugan CS, Blinkhorn AS (2005). Dental caries experience of British children in an international context. Community Dent Health.

[2] Bouwsma OJ (1996). The status, future, and problems of oral antiseptics. Curr Opin Periodontol.

[3] Naser-Alavi F, Salari A, Moein N, Talebzadeh A (2022). Effect of oral irrigation device and its solution type on the surface roughness and topography of Bulk-fill composite resins. J Clin Exp Dent.

[4] Alavi FN, Hajali F, Salari A, Moein N (2024). Effect of water and chlorhexidine with different pressures of oral irrigation device on the surface roughness and topography of giomer. Pesqui Bras Odontopediatria Clín Integr.

[5] Moein N, Alavi FN, Salari A, Mojtahedi A, Tajer A (2020). Effect of listerine mouthwash with green tea on the inhibition of streptococcus mutans: a microbiologic study. Pesqui Bras Odontopediatria Clín Integr.

[6] Addy M, Moran JM (1997). Clinical indications for the use of chemical adjuncts to plaque control: chlorhexidine formulations. Periodontol 2000.

[7] Wu CD, Savitt ED (2002). Evaluation of the safety and efficacy of over-the-counter oral hygiene products for the reduction and control of plaque and gingivitis. Periodontol 2000.

[8] He J, Wang S, Wu T, Cao Y, Xu X, Zhou X (2013). Effects of ginkgoneolic acid on the growth, acidogenicity, adherence, and biofilm of Streptococcus mutans in vitro. Folia Microbiol (Praha).

[9] Hirasawa M, Takada K, Otake S (2006). Inhibition of acid production in dental plaque bacteria by green tea catechins. Caries Res.

[10] Wen D, Liu Y, Li W, Liu H (2004). Separation methods for antibacterial and antirheumatism agents in plant medicines. J Chromatogr B Analyt Technol Biomed Life Sci.

[11] Kolouchová I, Maťátková O, Paldrychová M, Kodeš Z, Kvasničková E, Sigler K (2018). Resveratrol, pterostilbene, and baicalein: plant-derived anti-biofilm agents. Folia Microbiol (Praha).

[12] Abedini E, Khodadadi E, Zeinalzadeh E, Moaddab SR, Asgharzadeh M, Mehramouz B (2021). A Comprehensive Study on the Antimicrobial Properties of Resveratrol as an Alternative Therapy. Evid Based Complement Alternat Med.

[13] Garcia L, Garcia R, Pacheco G, Sutili F, Souza R, Mansur E (2016). Optimized Extraction of Resveratrol from Arachis repens Handro by Ultrasound and Microwave: A Correlation Study with the Antioxidant Properties and Phenol Contents. ScientificWorldJournal.

[14] Osamudiamen P, Oluremi B, Oderinlo O, Aiyelaagbe O (2020). Trans-resveratrol, piceatannol and gallic acid: Potent polyphenols isolated from Mezoneuron benthamianum effective as anticaries, antioxidant and cytotoxic agents. Scientific African.

[15] Li J, Wu T, Peng W, Zhu Y (2020). Effects of resveratrol on cariogenic virulence properties of Streptococcus mutans. BMC Microbiol.

[16] Nijampatnam B, Zhang H, Cai X, Michalek SM, Wu H, Velu SE (2018). Inhibition of Streptococcus mutans Biofilms by the Natural Stilbene Piceatannol Through the Inhibition of Glucosyltransferases. ACS Omega.

[17] Sekino S, Ramberg P (2005). The effect of a mouth rinse containing phenolic compounds on plaque formation and developing gingivitis. J Clin Periodontol.

[18] Haghgoo R, Mehran M, Zadeh HF, Afshari E, Zadeh NF (2017). Comparison Between Antibacterial Effect of Chlorhexidine 0.2% and Different Concentrations of Cyperus rotundus Extract: An In vitro Study. J Int Soc Prev Community Dent.

[19] Barbieri R, Coppo E, Marchese A, Daglia M, Sobarzo-Sánchez E, Nabavi SF (2017). Phytochemicals for human disease: An update on plant-derived compounds antibacterial activity. Microbiol Res.

[20] He Z, Huang Z, Zhou W, Tang Z, Ma R, Liang J (2016). Anti-biofilm Activities from Resveratrol against Fusobacterium nucleatum. Front Microbiol.

[21] Lee JH, Kim YG, Raorane CJ, Ryu SY, Shim JJ, Lee J (2019). The anti-biofilm and anti-virulence activities of trans-resveratrol and oxyresveratrol against uropathogenic Escherichia coli. Biofouling.

[22] Salehi B, Mishra AP, Nigam M, Sener B, Kilic M, Sharifi-Rad M (2018). Resveratrol: A Double-Edged Sword in Health Benefits. Biomedicines.

[23] Vestergaard M, Ingmer H (2019). Antibacterial and antifungal properties of resveratrol. Int J Antimicrob Agents.

[24] Pandit S, Kim HJ, Park SH, Jeon JG (2012). Enhancement of fluoride activity against Streptococcus mutans biofilms by a substance separated from Polygonum cuspidatum. Biofouling.

[25] Pirzada AM, Ali HH, Naeem M, Latif M, Bukhari AH, Tanveer A (2015). Cyperus rotundus L.: Traditional uses, phytochemistry, and pharmacological activities. J Ethnopharmacol.

[26] Cebrián R, Li Q, Peñalver P, Belmonte-Reche E, Andrés-Bilbao M, Lucas R (2022). Chemically Tuning Resveratrol for the Effective Killing of Gram-Positive Pathogens. J Nat Prod.

[27] Ferrazzano GF, Amato I, Ingenito A, Zarrelli A, Pinto G, Pollio A (2011). Plant polyphenols and their anti-cariogenic properties: a review. Molecules.

